# *PfmPif97-like* regulated by Pfm-miR-9b-5p participates in shell formation in *Pinctada fucata martensii*

**DOI:** 10.1371/journal.pone.0226367

**Published:** 2019-12-12

**Authors:** Xinwei Xiong, Bingyi Xie, Zhe Zheng, Yuewen Deng, Yu Jiao, Xiaodong Du

**Affiliations:** 1 Fishery College, Guangdong Ocean University, Zhanjiang, China; 2 Guangdong Technology Research Center for Pearl Aquaculture and Process, Guangdong Ocean University, Zhanjiang, China; University of Hong Kong, HONG KONG

## Abstract

Mollusk shell matrix proteins are important for the formation of organic frameworks, crystal nucleation, and crystal growth in *Pinctada fucata martensii* (*P*. *f*. *martensii*). MicroRNAs (miRNAs) are endogenous small non-coding RNAs that play important roles in many biological processes, including shell formation. In this study, we obtained the full-length sequence of *Pif97-like* gene in *P*. *f*. *martensii* (*PfmPif97-like*). *PfmPif97-like* was mainly distributed in mantle pallial and mantle edge. Correlation analysis indicated that the average shell thickness and weight showed a positive correlation with *PfmPif97-like* expression (*P < 0*.*05*). The inner surface of the nacreous layer and prismatic layer showed atypical growth when we knocked down the expression of *PfmPif97-like* by RNA interference (RNAi). We used a luciferase reporter assay to identify that miR-9b-5p of *P*. *f*. *martensii* (Pfm-miR-9b-5p) downregulated the expression of *PfmPif97-like* by interacting with the 3′-untranslated region (UTR) while we obtained the same result by injecting the Pfm-miR-9b-5p mimics in vivo. After injecting the mimics, we also observed abnormal growth in nacre layer and prismatic layer which is consistent with the result of RNAi. We proposed that *PfmPif97-like* regulated by Pfm-miR-9b-5p participates in shell formation of *P*. *f*. *martensii*. These findings provide important clues about the molecular mechanisms that regulate biomineralization in *P*. *f*. *martensii*.

## Introduction

Biomineralization is a special biological process that achieves precise regulation through organic matrix [1]. Mollusk shell is a stable organic mineral product consisting of calcium carbonate and organic matrices, including proteins, polysaccharides, and lipids [2, 3]. Shell matrix protein (SMP) is involved in nucleation, polymorphism, orientation, morphology, and organization of calcium carbonate crystallites in the shell [4]. Nacrein [5], MSI60 [6], N19 [7], N16 [8], Pif80 [9], Pif97 [9], and P10 [10] participate in nacre layer formation. MSI31 [6], MSI7 [11], aspein [12], prisilkin-39 [13], prismalin-14 [14], KRMP family [15], and prismin family [16] have key effects in prismatic layer. Shematrin family [17], PfY2 [18], and PNU9 [19] are involved in nacre and prismatic layers. SMPs are basically clear according to the shell proteome and genome [20]. Pif is a matrix protein consisting of Pif97 and Pif80 [9]. Suzuki identified Pif homologs from *Pinctada margaritifera*, *Pinctada maxima*, *Pteria penguin*, and *Mytilus galloprovincialis*, and found that Pif homologs from mollusks and gastropods contain more conserved von Willebrand factor type A domain (VWA) and chitin-binding domain (ChtBD) [9, 21]. Pif97 is involved in the calcification of nacre, including the binding of inorganic phase and polysaccharide template [22]. The recombinant protein Pif97 could interact with the recombinant protein N16.3 to form macromolecules under the action of calcium ions [23]. CgPif97 participates in shell formation in Pacific oysters [24]. Multiple pieces of evidence have denoted the importance of matrix protein containing VWA and ChtBD.

SMPs are not only participants of organic framework but also crystal regulators [9]. On vertebrates, miRNAs act as regulators of extracellular accumulation, osteoclast and osteoblast differentiation, transcription factor expression, and growth factor secretion [25]. miRNA regulators exist not only in vertebrates but also in invertebrates. Several miRNAs in mollusk were identified by using Solexa deep sequencing or bioinformatics analysis [26]. miR-29a [27], miR-183 [28], and miR-2305 [29] are involved in shell formation via the downregulation of matrix protein gene expression. These pieces of evidence signify that miRNAs commonly regulate the matrix protein gene to participate in shell formation.

Although some SMPs have been identified, the detailed molecular mechanisms of shell biomineralization remain poorly understood. In this study, we identified a matrix protein gene *PfmPif97-like* and focused on the function of biomineralization and miRNA regulation.

## Materials and methods

### Experimental materials

The experimental animals *P*. *f*. *martensii* (5–6 cm shell length) were sampled from Liushawan, Zhanjiang, in the South China Sea. The animals were temporarily farmed with circulating seawater until use.

### RNA isolation, cDNA synthesis, gene cloning, and real-time PCR assay

Total RNA was prepared using TRIzol reagent (Invitrogen, Carlsbad, CA, USA) according to the manufacturer’s instructions with some modification (https://dx.doi.org/10.17504/protocols.io.9qgh5tw) and cDNA was synthesized by M-MLV reverse transcriptase (Promega, USA). miRNAs were extracted by using SanPrep Column microRNA Extraction Kit (Sangon Biotech) and miRNA First-Strand was synthesized by using Mir-X miRNA First-Strand Synthesis Kit (TaKaRa). The 3′ and 5′ ends of the *PfmPif97-like* gene were cloned by using rapid amplification of cDNA ends (RACE) technology. The expression level was detected by Real-time PCR (RT-PCR) with DyNAmo Flash SYBR Green qPCR kit (Thermo Scientific) on a Roche LightCycler 480 (Roche, Switzerland). The PCR program was conducted as follows: 5 min at 95°C and 40 cycles (each cycle was for 30 s at 95°C, 15 s at 60°C, and 15 s at 72°C) The relative expression level of the target genes was calculated through the 2 ^(CT *β-actin—*CT Target gene)^ method, and β-actin was used as the reference gene. All primers and mimics sequences used in this study are listed in Table 1.

**Table 1:** The primers and sequence used in the study.

Primer	Sequence	Application
PfmPif97-like-S	ATGGGTATAGTTGTCTACAGCAGCA	CDS
PfmPif97-like-A	TTATCTAAGATGTGTAGGACGACACATG	CDS
PfmPif97-like-478-5'	CAGAGATTGGTGCCTGCGTGGGTG	RACE
PfmPif97-like-706-5'	GGTAGGAGAGTAATCTGGGATGGCGGC	RACE
PfmPif97-like-1174-3'	GCCAATGTGGAGCACTACTCGGAC	RACE
PfmPif97-like-1418-3'	AGAAGGCAAAGTaAATGGAATAGGGATG	RACE
pmiR-UTR-S	ggactagtccAACACACTGGTCAACCCAATCAT	subclone
pmiR-UTR-A	cccaagcttgggAGAGGCGACATCCATTCAAAAG	subclone
Pfm-miR-9b-5p	UCUUUGGUUACCUAGCUGUAUGA	mimics
microRNA N. C.	UUCUCCGAACGUGUCACGU	mimics
RT-PfmPif97-like-S	CAAGCCCCAGACCAGGAGTT	RT-PCR
RT-PfmPif97-like-A	CAGAGGACGCAATGCCGAT	RT-PCR
U6 Forward	GGAACGATACAGAGAAGATTAGC	RT-PCR
U6 Reverse	TGGAACGCTTCACGAATTTGCG	RT-PCR
RTPfm-miR-9b-5p	TCTTTGGTTACCTAGCTGTATGA	RT-PCR
NUP	AAGCAGTGGTATCAACGCAGAGT	RACES
UPM-Short	CTAATACGACTCACTATAGGGC	RACE
UPM-Long	CTAATACGACTCACTATAGGGCAAGCAGTGGTATCAACGCAGAGT	RACE
ISH- PfmPif97-like-S	CCATCCCAGATTACTCTCCTACC	ISH
ISH- PfmPif97-like-A	TAATACGACTCACTATAGGGCCCATCCAAAACCATACACG	ISH

The bases of lowercase letters are restriction enzyme sites. The underlined bases are the T7 promoter sequence.

### Sequence analysis and target gene prediction

The open reading frame (ORF) was obtained using the ORF Finder tool (https://www.ncbi.nlm.nih.gov/orffinder/). The protein domain was predicted by using the Simple Modular Architecture Research Tool (http://smart.embl-heidelberg.de/smart/show_motifs.pl). Multiple sequence alignment was performed with Clustal Omega website tool (https://www.ebi.ac.uk/Tools/msa/clustalo/). Target prediction between Pfm-miR-9b-5p and *PfmPif97-like* was performed using miRanda [30] and RNAhybrid [31] software.

### Expression distribution pattern of *PfmPif97-like* and Correlation analysis

In situ hybridization was used to determine the expression distribution of *PfmPif97-like* in the mantle. RNA probes were prepared by in vitro transcription using T7 RNA polymerase and digoxigenin (DIG) RNA Labeling Mix. RNA probe integrity was detected by using 1% agarose gel electrophoresis, and the quality of probes was analyzed in conjunction with the RNA concentration and purity determined by using a nucleic acid quantifier. The mantle tissues were fixed with 4% paraformaldehyde for 2 hours at 4°C. Fixative volume is over 20 times that of tissue on a weight per volume. Then the tissue was dehydrated through a series of graded ethanol baths to displace the water, and then infiltrated with wax. The infiltrated tissues were then embedded into wax blocks. Then the mantle tissue were cut into 7 μm in thickness via the instrument of LEICA RM2235. In situ hybridization was carried out according to the instructions of Enhanced Sensitive ISH Detection Kit I (POD) (BOSTER) with some modification. This protocol has been deposited in protocols.io (dx.doi.org/10.17504/protocols.io.9qhh5t6).

Thirty-five normal pearl oysters were select to perform the notching assays. Thirty normal pearl oysters were select and cut a “V” shaped notch until the nacreous layer of the shell. Collected the mantle edge of every five pearl oysters at 2h, 4h, 6h,12h, 24h and 36h after damage and harvested the mantle edge of five pearl oysters (no notching) at 0 h. The larval sample is the same sample as the sample of the developmental transcriptome in the previous genomic article.

The Pearson correlation coefficient between *PfmPif97-like* expression in mantle edge and growth traits was examined using Pearson test in SPSS 22.0. A total number of 21 normal pearl oyster were used for Pearson correlation coefficient analysis. Shell parameter contains shell length, shell width, shell height, shell weight and shell thickness.

### *PfmPif97-like* function interference experiment in vivo

Double-stranded RNA (dsRNA) was synthesized using the T7 High-Efficiency Transcription Kit (TransGen Biotech, JT101) and purified using EasyPure RNA Purification Kit (TransGen Biotech, ER701). Fifteen *P*. *f*. *martensii* individuals of similar size (5–6 cm shell length) were randomly divided into three groups. Double-stranded RNA (dsRNA) (60 μg/100 μL) and diethyl pyro carbonate (DEPC) water were injected into the adductor muscle. 100 μL of dsRNA-PfmPif97-like was injected as the experimental group. For control groups, 100 μL of DEPC water was injected and 100 μL of dsRNA-Red Fluorescent Protein (RFP) was injected. On the sixth day after injection, the mantle tissue was collected, frozen rapidly in liquid nitrogen, and stored at −80°C. The shells were cut into 0.5 cm × 1.5 cm size and washed with ultrapure water. The shell samples were air dried, and the inner surface of the shell was observed by using scanning electron microscope (SEM) in 15 kV.

### Target verification between Pfm-miR-9b-5p and *PfmPif97-like in vitro* and *in vivo*

The *PfmPif97-like* 3′UTR containing the target site was cloned by using PrimeSTAR HS DNA Polymerase (purchased from takara). Then the sequence was inserted into a pMIR-reporter plasmid (purchased from Ambion) (pMi-UTR) by using restriction enzyme. We also constructed a mutant plasmid (pMi-MUTR) as the control. The Pfm-miR-9b-5p mimics and negative control mimics (N. C.) were compounded from GenePharma. The pMi-UTR and pMi-MUTR groups were blank control group. The N. C. and pMi-UTR co-transfection group, N. C. and pMi-MUTR co-transfection group, and Pfm-miR-9b-5p mimics and pMi-MUTR co-transfection group were negative control group. The Pfm-miR-9b-5p mimics and pMi-UTR co-transfection group was the experimental group. Each group was co-transfected with 22.5 ng of the pRL-TK vector as internal quality control to determine the relative activity in a dual-luciferase report system. Luciferase activity was detected using the dual-luciferase assay kit (Promega) with a microplate reader at 48 h after transfecting to HEK-293T cells cultured in 48-well plate. HEK-293T cells were cultured at 37°C in DMEM/HIGE GLUCOSE medium containing 10% fetal bovine serum in a CO_2_ incubator with 5% CO_2_.

Pfm-miR-9b-5p mimics and N. C. mimics were diluted to 0.1 μg/μL of RNase-free water, and 100 μL was injected into the adductor muscles. The N. C. group served as the negative control. Five *P*. *f*. *martensii* individuals (5–6 cm shell length) were used for each group. The mantle tissue and shell of each sample were collected correspondingly and the expression level was detected using RT-PCR. The inner surface of the nacre and prism layer was observed by using an SEM.

## Results

### Cloning and characterization of *PfmPif97-like*

**Fig 1:**
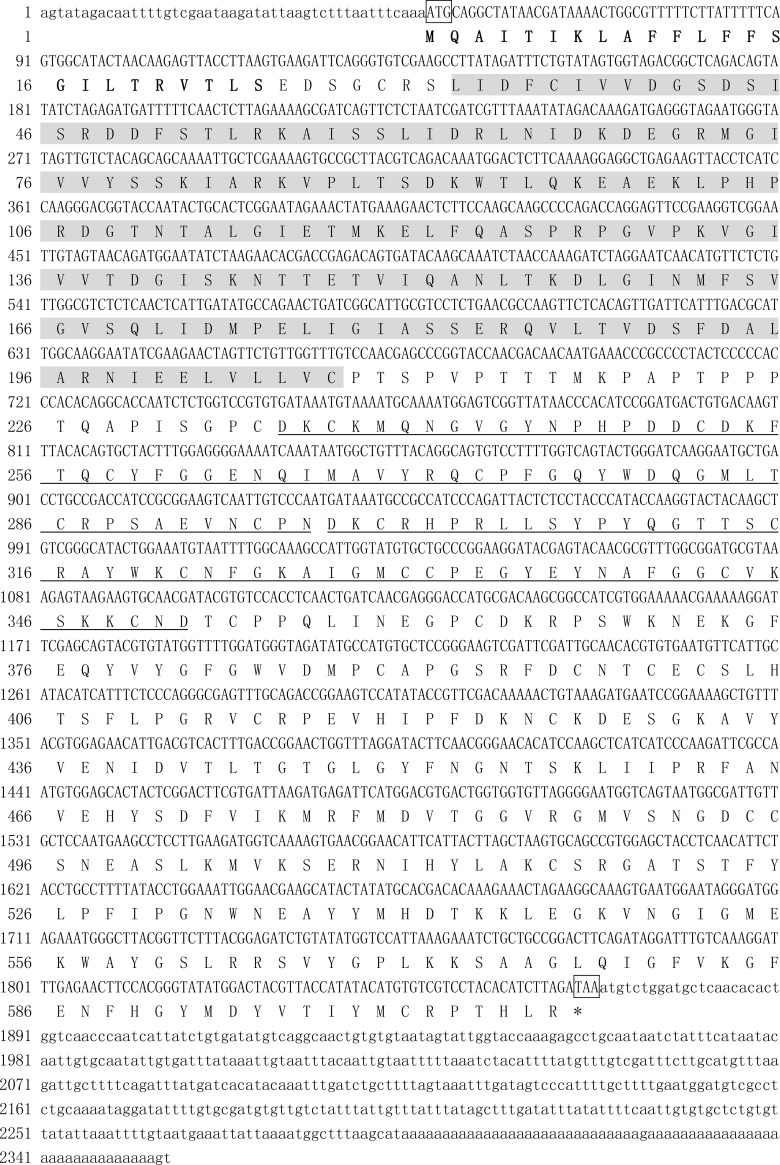
Nucleotide and amino acid sequence of *PfmPif97-like*. The amino acid sequences are shown below the nucleotide sequence. The amino acid sequence with the black bold font is signal peptide and with gray background is VWA domain. The underlined amino acid sequences are two ChtBD2 domains. The nucleotide with a frame represents the start and stop codons. The sequence before ATG is the 5′UTR region. The sequence after TAA is the 3′UTR region.

**Fig 2:**
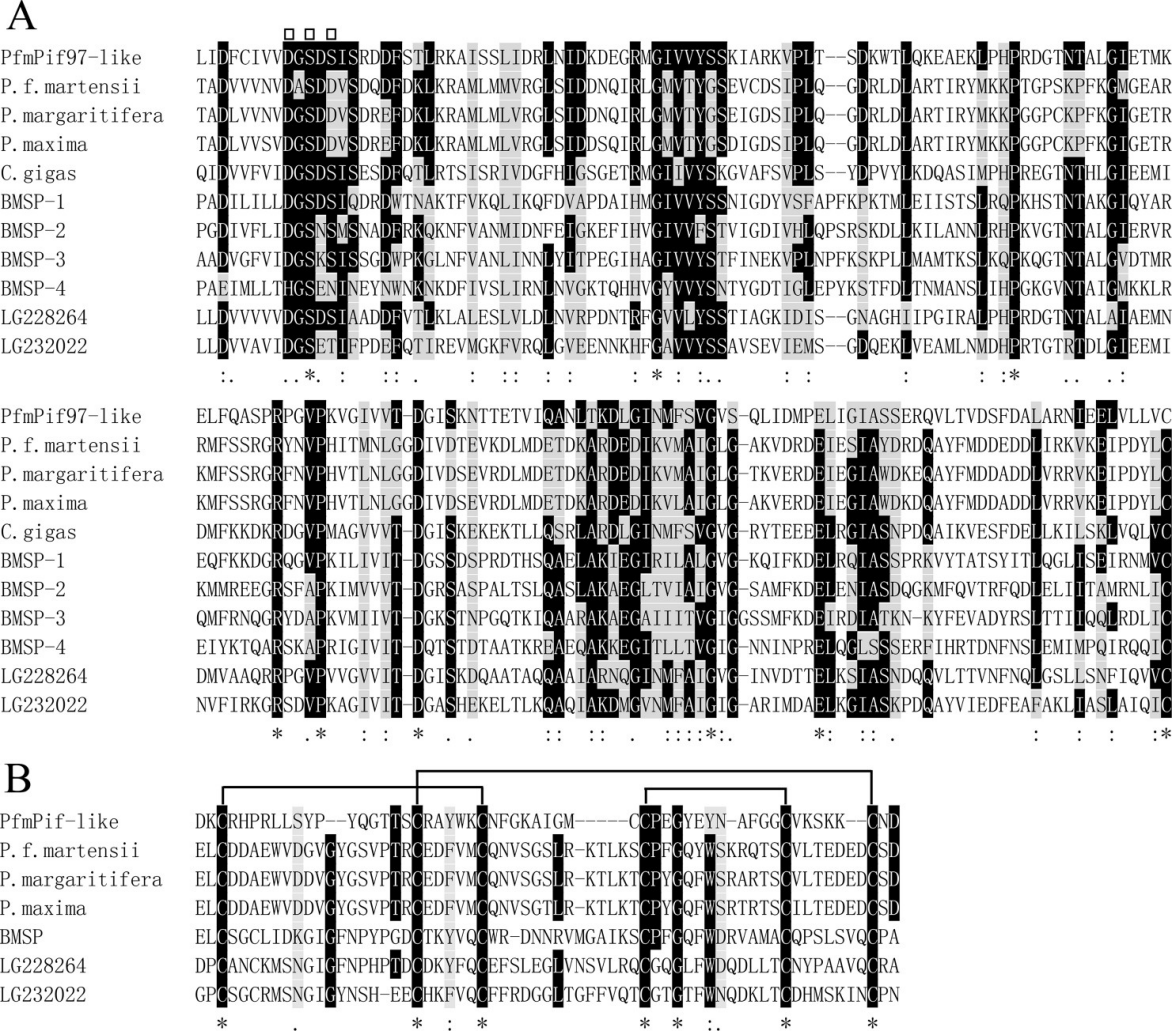
The alignment result of VWA and Chitin-binding domain. A: Amino acid sequence alignment of the VWA domains. B: Amino acid sequence alignment of the chitin binding domain. The amino acid marked with a square indicated the MIDAS motif, DXSXS. Wires indicate the completely conserved six cysteine residues among all chitin-binding domains. “*” showed the same amino acid. The black background indicates the conserved amino acid; “:” with a gray background shows the amino acid with strong similarity; “.” with a gray background indicates amino acid with weak similarity. *P*. *f*. *martensii*: *Pinctada fucata martensii*; *P*. *margaritifera*: *Pinctada margaritifera*; *P*. *maxima*: *Pinctada maxima*; *C*. *gigas*:; BMSP has four VWA domains, namely, BMSP-1, BMSP-2, BMSP-3, BMSP-4; LG228264 and LG232022 have one VWA domain.

The full-length cDNA of *PfmPif97-like* (GenBank accession numbers: MK962312) was 2356 bp, containing an 1821 bp ORF encoding a putative protein of 606 aa, 5′UTR of 47 bp, and 3′UTR of 458 bp (Fig 1). PfmPif97-like contains a VWA domain and two ChtBD type 2 (ChtBD2). The local BLASTp search indicated that the amino acid sequence of PfmPif97-like showed 26%–41% identity to those of PfmPif97, PmPif97, PmxPif97, PpPif97, and CgPif97 (S1 File). Sequence alignment of VWA and ChtBD2 domains from PfmPif97-like, Pif, and Pif homologs from several mollusks revealed the conservation functional site in the domain (Fig 2).

### Expression distribution, expression pattern and correlation analysis

**Fig 3:**
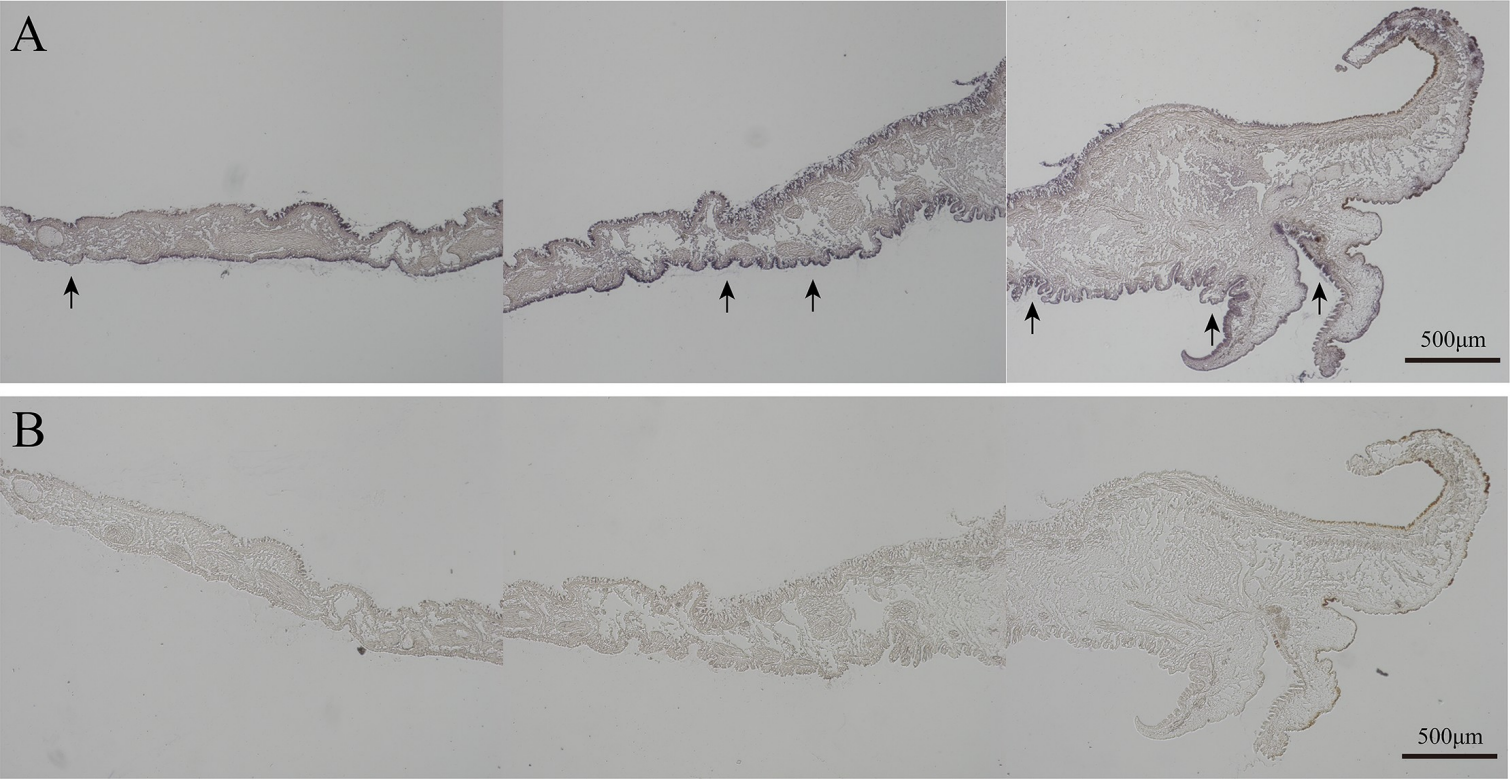
*In situ* hybridization results. The arrows indicate the positive signal. A: The picture of experimental group. The arrows are indicated the positive signal. B: The picture of control group. Black arrow indicates positive signal. The black line in the image indicates the scale.

**Fig 4:**
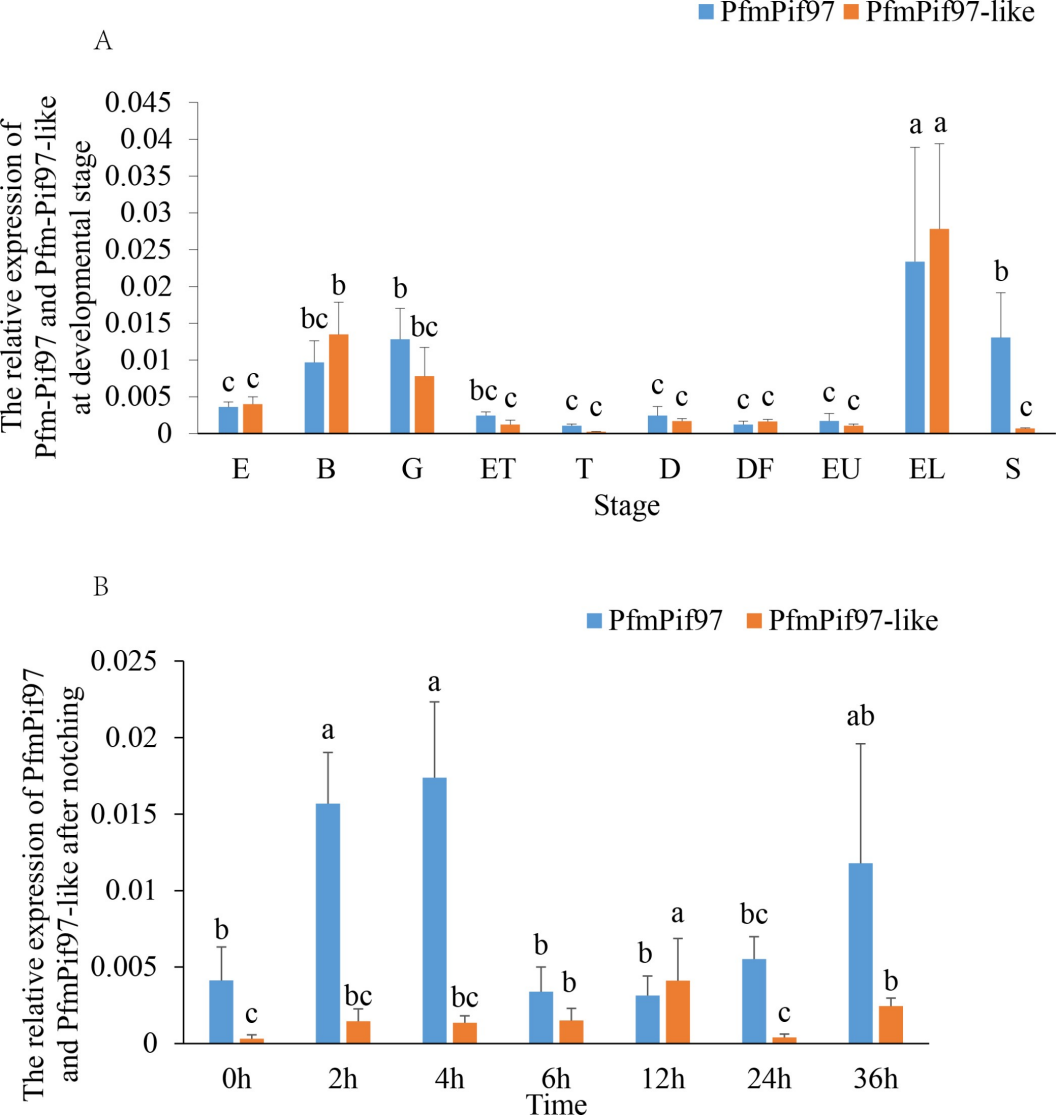
Expression patterns of *PfmPif97-like* and *PfmPif97*. A: Expression of *PfmPif97-like* and *PfmPif97* in larval developmental stage. B: Expression of *PfmPif97-like* and *PfmPif97* at different time points after notching. The averages of the groups with the same lower-case letters (a, b, c) were not significantly different. Abbreviations in picture A: E: egg; B: blastula; G: gastrula; ET: early trochophore; T: trochophore; D: D-shaped larvae; DF: D-shaped larvae before feeding; EU: Early umbo larvae; EL: eye-spotted larvae; S: post-veliger stage.

Result of in situ hybridization demonstrated that *PfmPif97-like* was mainly distributed in the mantle pallial, mantle edge and was little distributed in the central zone of mantle (Fig 3). *PfmPif97* expression was significantly increased at 2 h, 4 h and 36 h after shell damage (Fig 4A) while *PfmPif97-like* expression was significantly increased at 6 h, 12 h and 36 h (Fig 4A). In larval development stage, *PfmPif97* was highly expressed in gastrula, eye-spotted larvae and post-veliger stage (Fig 4B) while *PfmPif97-like* were highly expressed in blastula and eye-spotted larvae (Fig 4B).

The Pearson correlation coefficient showed *PfmPif97-like* expression exhibited significant correlation with wet shell weight (R = 0.753, *P < 0*.*05*), dry shell weight (R = 0.762, *P < 0*.*05*), the average thickness of the left shell (R = 0.751, *P < 0*.*05*) and the average thickness of the right shell (R = 0.762, *P < 0*.*05*) (Table 2). However, no significant correlation was observed between *PfmPif97-like* expression and shell width, height, and length.

**Table 2:** Correlation analysis between *PfmPif97-like* expression and growth traits of *P*. *f*. *martensii*.

		Shell length	Shell width	Shell height	Total Weight	Wet shell weight	Dry shell weight	Average thickness of the left shell	Average thickness of the right shell
*PfmPif97-like*	*R*	0.204	0.135	-0.124	0.258	0.753**	0.762**	0.751**	0.762**
Expression levels	*P*	0.484	0.646	0.672	0.373	0.002	0.002	0.002	0.002

The number in the table indicated the correlation coefficient (R), R>0 showed positive correlation and R<0 presented a negative correlation. Correlations with “**” showed significant (P<0.01).

### *PfmPif97-like* interference disrupted shell biomineralization

**Fig 5:**
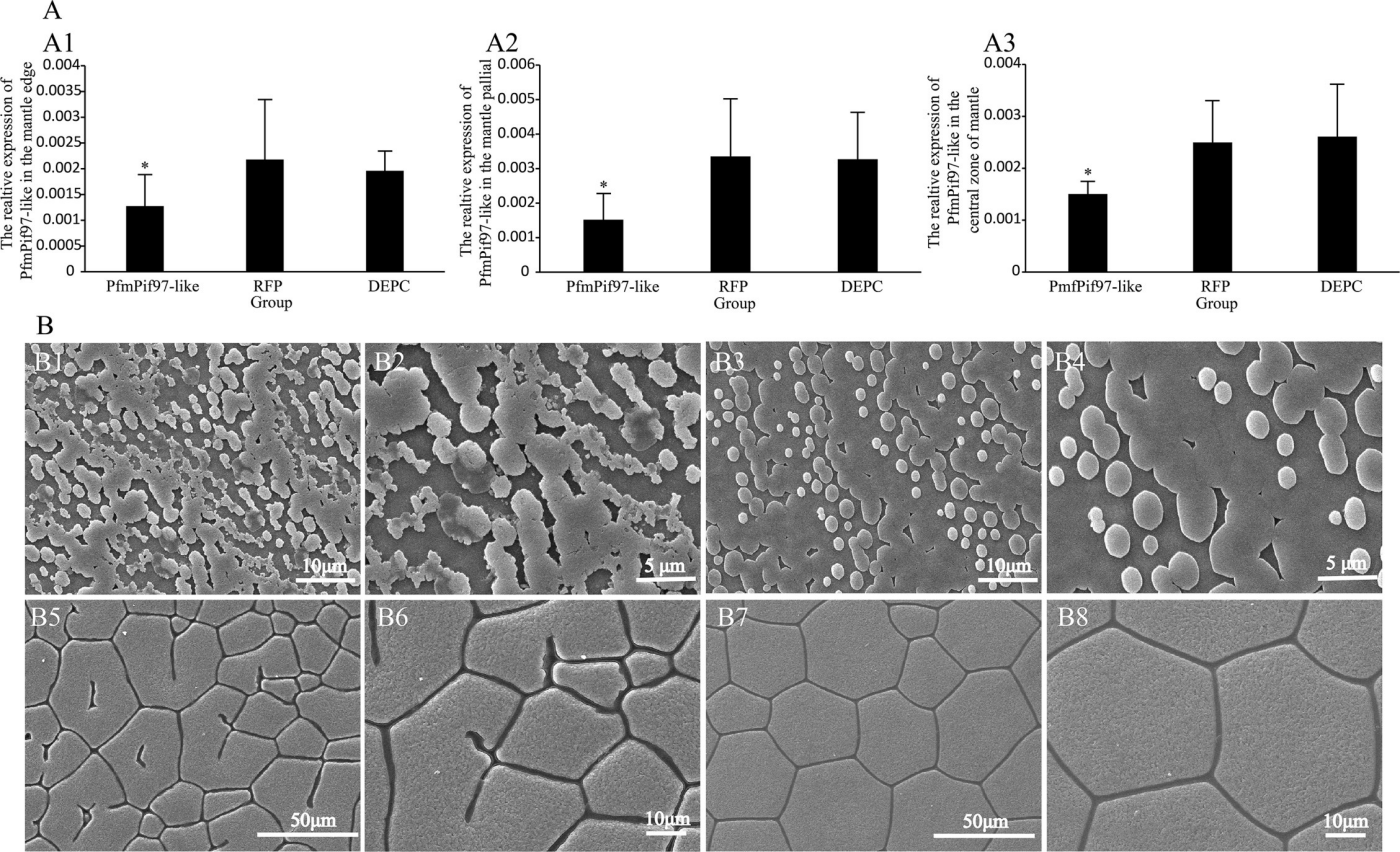
The relative expression of *PfmPif97-like* and SEM images of nacre and prismatic layer after injecting dsRNA. A: The relative expression of *PfmPif97-like* following inhibition in mantle edge (A1); mantle pallial (A2); the central zone of mantle (A3). “*” means a significant difference (*P* < *0*.*05*). B: B1 and B2: SEM image of the nacre layer of experimental group. B3 and B4: SEM image of the nacre layer of control group. B5 and B6: SEM image of the prismatic layer of experimental group. B7 and B8: SEM image of the prismatic layer of control group. The white line in the image indicates the scale.

The interference experiment showed *PfmPif97-like* expression levels in the mantle pallial, mantle edge and central zone of mantle were significantly downregulated compared with those of the control group (*P < 0*.*05*) (Fig 5A). The nacre of the corresponding shell grew disorderly and slowly (Fig 5B), and the prismatic layer changed from angular to fragmented (Fig 5B).

### Pfm-miR-9b-5p negatively regulated *PfmPif97-like* expression

**Fig 6:**
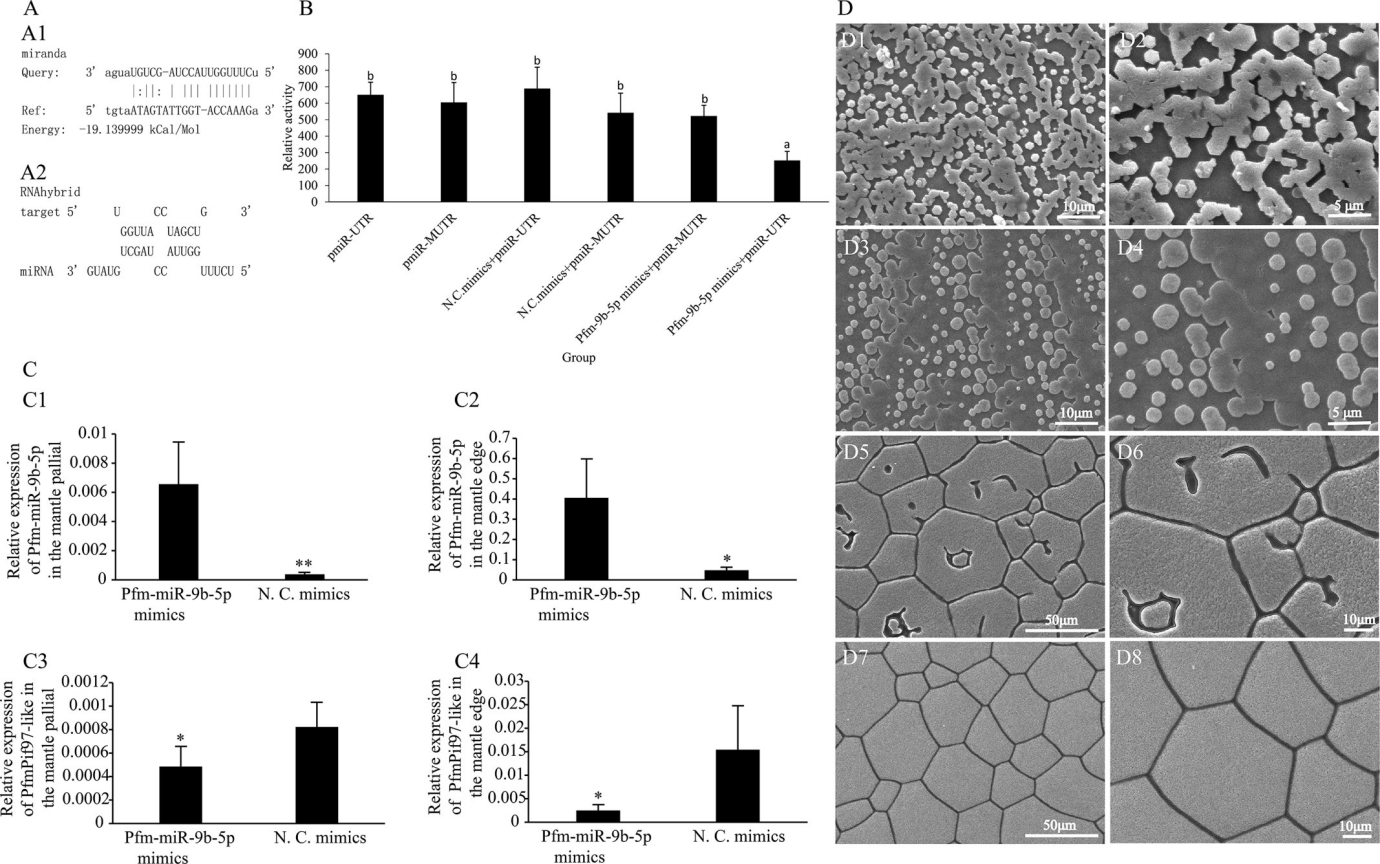
Pfm-miR-9b-5p targeting *PfmPif97-like* 3′UTR. A1 and A2: The target site obtained by using miranda and RNAhybrid. B: Results of dual-luciferase assay in 293T cells. The relative expression of Pfm-miR-9b-5p in the central zone of mantle (C1) and the mantle edge (C2) after injecting Pfm-miR-9b-5p mimics. The relative expression of *Pfm-Pif97-like* in the mantle pallial (C3) and the mantle edge (C4) after injecting Pfm-miR-9b-5p mimics. D1 and D2 SEM image of nacre layer after injecting Pfm-miR-9b-5p mimics; D3 and D4: SEM images of nacre layer after injecting N. C. mimics; D5 and D6: SEM images of prismatic layer after injecting Pfm-miR-9b-5p mimics; D7 and D8: SEM image of prismatic layer after injecting N. C. mimics. “a” and “*” represents a significant difference (*P < 0*.*05*); “b” represents no significant difference. The white line in the image indicates the scale.

The potential target site between the 3′UTRs of *PfmPif97-like* and Pfm-miR-9b-5p was obtained by using miRanda and RNAhybrid software (Fig 6A). In vitro, luciferase activity was downregulated in Pfm-miR-9b-5p mimics and pMi-UTR co-transfection group compared with that in the control groups (*P < 0*.*05*) (Fig 6B). The expression of Pfm-miR-9b-5p was significantly upregulated after injecting the Pfm-miR-9b-5p mimics, whereas that of *PfmPif97-like* was significantly downregulated (Fig 6C). The surface of the nacre and prism layer was disordered (Fig 6D).

## Discussion

SMPs are considered structural and functional macromolecules [6, 7, 11]. The function of matrix protein in shell formation has been extensively studied, including nacrein [5], Pif [9], ACCBP [32], PNU7 [19], Pfy2 [18], and N19 [7]. However, the mechanism of shell formation is not fully understood. In this study, we obtained the full-length sequence of *PfmPif97-like* which is similar to PfmPif97. Whether in the nacre or in the prismatic layer, chitin serves as the key component of the organic framework [3], building compartment structures and linking with matrix protein to the morphology of calcium carbonate crystals [33,34]. In addition to chitinase [35], the matrix protein with the chitin-binding domain is related to chitin [22]. The chitin-binding domain is an extracellular domain that contains six conserved cysteines that probably form three disulfide bridges [36], which are also found in PfmPif97-like. The recombinant PfmPif97 is a framework protein for the association of chitin-aragonite [22]. Similar to PfmPif97, PfmPif97-like has two ChtBD2, indicating that PfmPif97-like may be involved in the formation of the organic framework by binding β-chitin. PfmPif97-like also has a VWA domain which is a family of 200-amino-acid residues and works as an interaction module [37]. VWA-containing proteins are widely found in *P*. *margaritifera* [38], *Mytilus coruscus* [39], *Crassostrea gigas* [40], *Lottia gigantea* [41], and *P*. *f*. *martensii* [20]. In molluscks, VWA-containing proteins may be involved in protein-protein interactions, providing initial hydrogel properties for biomineralization [20, 37]. Thus, PfmPif97-like may act as a medium for connecting chitin and SMP like PfmPif97.

We found PfmPif97-like was mainly expressed in the mantle pallial, mantle edge and had a small distribution in the central zone of mantle, which are regions responsible for the nacreous layer and prismatic layer formation. And the *PfmPif97-like* expression level in mantle edge was significantly correlated with shell weight and thickness. This showed that PfmPif97-like may participate in oyster shell formation. *PfmPif97* expression was significantly increased at 2 h, 4 h, and 36 h after shell notching. However, *PfmPif97-like* expression was significantly increased at 6 h, 12 h and 36 h. The expression pattern of *PfmPif97-like* in the notching experiment is similar to that of alveoline-like protein [42], which plays essential role in shell formation. Shell notching causes mantle tissue retraction, causing an immune response and disturbing shell protein secretion.[43]. After that, the shell regeneration process is mainly carried out [43]. Thus, PfmPif97-like and PfmPif97 participate in shell regeneration, and Pfmpif97-like might be less related to immune response after shell notching than PfPif97. *PfmPif97-like* were highly expressed in the blastula and *PfmPif97* was highly expressed in the gastrula. This indicates that they have the different function at the early larval development stage. Li found that a large number of genes involved in the calcium signaling pathway and synchronization with the shell formation were upregulated during the eye-spotted stage [44]. Thus, *PfmPif97-like* and *PfmPif97* are highly expressed in the eye-spotted larvae suggesting that they play a role in shell formation. To directly determine whether PfmPif97-like is involved in shell formation, we knocked down the expression level of *PfmPif97-like* by using RNAi and found that the nacre and prism layers grew disorderly. Therefore, PfmPif97-like participates in shell formation, possibly by linking chitin and other SMPs.

Mollusk shell formation is a complex process that requires precise regulation [3, 26]. miRNA is a negative regulator [45]. miRNAs target approximately 60% of genes of mammals [46] showing their important biological functions. miRNAs, such as miR-29a, miR-183, and miR-2305, were found to participate in shell formation by targeting biomineralization genes [27–29]. These show that miRNAs are generally involved in shell formation. In the present study, we found that Pfm-miR-9b-5p may be targeting *PfmPif97-like* 3′UTR, and the relationship of regulation was verified by dual-luciferase system in vitro. In vivo, upon the overexpression of Pfm-miR-9b-5p via injection with Pfm-miR-9b-5p mimics, the expression of *PfmPif97-like* was downregulated, and the nacre and prism layers exhibited atypical growth, similar to the results of RNAi. These show that PfmPif97-like is downregulated by Pfm-miR-9b-5p and participates in shell formation.

## Conclusion

We discover a matrix protein gene similar to *PfmPif97*, named *PfmPif97-like*, from *P*. *f*. *martensii* and we ascertained that PfmPif97-like is regulated by Pfm-miR-9b-5p and participates in shell formation, possibly by linking chitin and other SMPs. These findings provide important clues about the molecular mechanisms that regulate biomineralization in *P*. *f*. *martensii*.

## Supporting information

S1 File The output result of local BLASTp. The version of the local BLASTp program is BLASTP 2.7.1+. The database is the Pif97 sequence collected from NCBI.
(DOCX)
