# Supplementary material for: PfmPif97-like regulated by Pfm-miR-9b-5p participates in shell formation in Pinctada fucata martensii
Source: PLoS One. 2019 Dec 12;14(12):e0226367. doi: 10.1371/journal.pone.0226367 (PMC6907788; doi:10.1371/journal.pone.0226367)
Supplement: S1 File — The version of the local BLASTp program is BLASTP 2.7.1+. The database is the Pif97 sequence collected from NCBI. (DOCX) [file pone.0226367.s001.docx]

BLASTP 2.7.1+

Reference: Stephen F. Altschul, Thomas L. Madden, Alejandro A.

Schaffer, Jinghui Zhang, Zheng Zhang, Webb Miller, and David J.

Lipman (1997), "Gapped BLAST and PSI-BLAST: a new generation of

protein database search programs", Nucleic Acids Res. 25:3389-3402.

Reference for composition-based statistics: Alejandro A. Schaffer,

L. Aravind, Thomas L. Madden, Sergei Shavirin, John L. Spouge, Yuri

I. Wolf, Eugene V. Koonin, and Stephen F. Altschul (2001),

"Improving the accuracy of PSI-BLAST protein database searches with

composition-based statistics and other refinements", Nucleic Acids

Res. 29:2994-3005.

Database: Pif97.fa

7 sequences; 3,889 total letters

Query= PfmPif97 like(Pinctada fucata martensii)

Length=606

Score E

Sequences producing significant alignments: (Bits) Value

PfmPif97 like(Pinctada fucata martensii) unnamed protein product 1264 0.0

LgPif97(XP_009051492.1Lottia gigantea) unnamed protein product 366 9e-123

CgPif97(AFT63505.1Crassostrea gigas) unnamed protein product 335 5e-112

PfmPif97(Pinctada fucata martensii) unnamed protein product 217 1e-066

PpPif97(BAM66824.1Pteria penguin) unnamed protein product 206 4e-063

PmxPif97(BAJ08001.1Pinctada maxima) unnamed protein product 204 1e-061

PmPif97(BAM66823.1Pinctada margaritifera) unnamed protein product 202 3e-061

>PfmPif97 like(Pinctada fucata martensii) unnamed protein product

Length=606

Score = 1264 bits (3271), Expect = 0.0, Method: Compositional matrix adjust.

Identities = 606/606 (100%), Positives = 606/606 (100%), Gaps = 0/606 (0%)

Query 1 MQAITIKLAFFLFFSGILTRVTLSEDSGCRSLIDFCIVVDGSDSISRDDFSTLRKAISSL 60

MQAITIKLAFFLFFSGILTRVTLSEDSGCRSLIDFCIVVDGSDSISRDDFSTLRKAISSL

Sbjct 1 MQAITIKLAFFLFFSGILTRVTLSEDSGCRSLIDFCIVVDGSDSISRDDFSTLRKAISSL 60

Query 61 IDRLNIDKDEGRMGIVVYSSKIARKVPLTSDKWTLQKEAEKLPHPRDGTNTALGIETMKE 120

IDRLNIDKDEGRMGIVVYSSKIARKVPLTSDKWTLQKEAEKLPHPRDGTNTALGIETMKE

Sbjct 61 IDRLNIDKDEGRMGIVVYSSKIARKVPLTSDKWTLQKEAEKLPHPRDGTNTALGIETMKE 120

Query 121 LFQASPRPGVPKVGIVVTDGISKNTTETVIQANLTKDLGINMFSVGVSQLIDMPELIGIA 180

LFQASPRPGVPKVGIVVTDGISKNTTETVIQANLTKDLGINMFSVGVSQLIDMPELIGIA

Sbjct 121 LFQASPRPGVPKVGIVVTDGISKNTTETVIQANLTKDLGINMFSVGVSQLIDMPELIGIA 180

Query 181 SSERQVLTVDSFDALARNIEELVLLVCPTSPVPTTTMKPAPTPPPTQAPISGPCDKCKMQ 240

SSERQVLTVDSFDALARNIEELVLLVCPTSPVPTTTMKPAPTPPPTQAPISGPCDKCKMQ

Sbjct 181 SSERQVLTVDSFDALARNIEELVLLVCPTSPVPTTTMKPAPTPPPTQAPISGPCDKCKMQ 240

Query 241 NGVGYNPHPDDCDKFTQCYFGGENQIMAVYRQCPFGQYWDQGMLTCRPSAEVNCPNDKCR 300

NGVGYNPHPDDCDKFTQCYFGGENQIMAVYRQCPFGQYWDQGMLTCRPSAEVNCPNDKCR

Sbjct 241 NGVGYNPHPDDCDKFTQCYFGGENQIMAVYRQCPFGQYWDQGMLTCRPSAEVNCPNDKCR 300

Query 301 HPRLLSYPYQGTTSCRAYWKCNFGKAIGMCCPEGYEYNAFGGCVKSKKCNDTCPPQLINE 360

HPRLLSYPYQGTTSCRAYWKCNFGKAIGMCCPEGYEYNAFGGCVKSKKCNDTCPPQLINE

Sbjct 301 HPRLLSYPYQGTTSCRAYWKCNFGKAIGMCCPEGYEYNAFGGCVKSKKCNDTCPPQLINE 360

Query 361 GPCDKRPSWKNEKGFEQYVYGFGWVDMPCAPGSRFDCNTCECSLHTSFLPGRVCRPEVHI 420

GPCDKRPSWKNEKGFEQYVYGFGWVDMPCAPGSRFDCNTCECSLHTSFLPGRVCRPEVHI

Sbjct 361 GPCDKRPSWKNEKGFEQYVYGFGWVDMPCAPGSRFDCNTCECSLHTSFLPGRVCRPEVHI 420

Query 421 PFDKNCKDESGKAVYVENIDVTLTGTGLGYFNGNTSKLIIPRFANVEHYSDFVIKMRFMD 480

PFDKNCKDESGKAVYVENIDVTLTGTGLGYFNGNTSKLIIPRFANVEHYSDFVIKMRFMD

Sbjct 421 PFDKNCKDESGKAVYVENIDVTLTGTGLGYFNGNTSKLIIPRFANVEHYSDFVIKMRFMD 480

Query 481 VTGGVRGMVSNGDCCSNEASLKMVKSERNIHYLAKCSRGATSTFYLPFIPGNWNEAYYMH 540

VTGGVRGMVSNGDCCSNEASLKMVKSERNIHYLAKCSRGATSTFYLPFIPGNWNEAYYMH

Sbjct 481 VTGGVRGMVSNGDCCSNEASLKMVKSERNIHYLAKCSRGATSTFYLPFIPGNWNEAYYMH 540

Query 541 DTKKLEGKVNGIGMEKWAYGSLRRSVYGPLKKSAAGLQIGFVKGFENFHGYMDYVTIYMC 600

DTKKLEGKVNGIGMEKWAYGSLRRSVYGPLKKSAAGLQIGFVKGFENFHGYMDYVTIYMC

Sbjct 541 DTKKLEGKVNGIGMEKWAYGSLRRSVYGPLKKSAAGLQIGFVKGFENFHGYMDYVTIYMC 600

Query 601 RPTHLR 606

RPTHLR

Sbjct 601 RPTHLR 606

>LgPif97(XP_009051492.1Lottia gigantea) unnamed protein product

Length=606

Score = 366 bits (940), Expect = 9e-123, Method: Compositional matrix adjust.

Identities = 243/592 (41%), Positives = 331/592 (56%), Gaps = 30/592 (5%)

Query 23 LSEDSGCRSLIDFCIVVDGSDSISRDDFSTLRKAISSLIDRLNIDKDEGRMGIVVYSSKI 82

+S + C+SL+D +VVDGSDSI+ DDF TL+ A+ SL+ LN+ D R G+V+YSS I

Sbjct 31 ISSYTECKSLLDVVVVVDGSDSIAADDFVTLKLALESLVLDLNVRPDNTRFGVVLYSSTI 90

Query 83 ARKVPLTSDKWTLQKEAEKLPHPRDGTNTALGIETMKELFQASPRPGVPKVGIVVTDGIS 142

A K+ ++ + + LPHPRDGTNTAL I M ++ A RPGVP VG+V+TDGIS

Sbjct 91 AGKIDISGNAGHIIPGIRALPHPRDGTNTALAIAEMNDMVAAQRRPGVPVVGVVITDGIS 150

Query 143 KNTTETVIQANLTKDLGINMFSVGVSQLIDMPELIGIASSERQVLTVDSFDALARNIEEL 202

K+ T QA + ++ GINMF++GV +D EL IAS+++QVLT +F+ L +

Sbjct 151 KDQAATAQQAAIARNQGINMFAIGVGINVDTTELKSIASNDQQVLTTVNFNQLGSLLSNF 210

Query 203 VLLVCPTSPVPTTTMKPAPTPPPTQAPISG----------PCDKCKMQNGVGYNPHPDDC 252

+ +VCPT+ TTT T P + PC CKM NG+G+NPHP DC

Sbjct 211 IQVVCPTTTTSTTTTTTTTTTPAPTTSTTVPTTTTTVKPDPCANCKMSNGIGFNPHPTDC 270

Query 253 DKFTQCYFGGENQIMAVYRQCPFGQYWDQGMLTCRPSAEVNCPNDKCRHPRLLSYPYQGT 312

DK+ QC F E + +V RQC G +WDQ +LTC A V C D C++ + SY G

Sbjct 271 DKYFQCEFSLEGLVNSVLRQCGQGLFWDQDLLTCNYPAAVQCRADPCQNYHISSYKKAG- 329

Query 313 TSCRAYWKCNFGKAIGMCCPEGYEYNAFGGCVKSKKCNDTCPPQLINEGPCDKRPSWKNE 372

+CR Y+ C+ G ++ CC +G+ Y G CV S CN C IN C+ R +

Sbjct 330 -NCREYYSCSNGTSMPECCKKGFAY-VSGQCVPSYNCNAHCKGDFINP-YCEMRAVSDDI 386

Query 373 KGFEQYVYGFGWVDMPCAPGSRFDCNTCECSLHTSFLPGRV--CRPEVHIPFDKNCK-DE 429

+EQ+V G GWV PCAPGS F C C++ LP C+ EV+IPFD + D+

Sbjct 387 SSYEQFVRGVGWVRKPCAPGSAFSPVECSCTVAIDPLPINAGECKAEVYIPFDDDVAIDK 446

Query 430 SGKAVYVENIDVTLTGTGLGYFNGNTSKLIIPRFANVEHYSDFVIKMRF-MDVTGGVRGM 488

SG YVEN V + G G GYFNG TS L IPRF+N+E S VI MR+ + G +G+

Sbjct 447 SGNGNYVENEGVFVIG-GKGYFNG-TSGLRIPRFSNIEFGSKVVITMRYKAESIYGSQGL 504

Query 489 VSNGDCCSNEASLKMVKSERNIHYLAKCSRGATSTFYLPFIPGNWNEAYYMHDTKKLEGK 548

+SNGD C SL + N + + G +P G WNE Y + L G

Sbjct 505 ISNGD-CGKPGSLLVAIDNTNTLFGLQTVSGTAGIVTIPSANG-WNEIIYQVEGDVLTGS 562

Query 549 VNGIGMEKWAYGSLRRSVYGPLKKSAAGLQIGFVKGFENFHGYMDYVTIYMC 600

VNG S +++ G +K+S LQ+G NF GY+D +T+Y+C

Sbjct 563 VNG--------NSAHKTIDGAVKRSQCALQVGRATHLSNFRGYVDELTVYLC 606

>CgPif97(AFT63505.1Crassostrea gigas) unnamed protein product

Length=510

Score = 335 bits (860), Expect = 5e-112, Method: Compositional matrix adjust.

Identities = 216/578 (37%), Positives = 302/578 (52%), Gaps = 109/578 (19%)

Query 28 GCRSLIDFCIVVDGSDSISRDDFSTLRKAISSLIDRLNIDKDEGRMGIVVYSSKIARKVP 87

GC++ ID V+DGSDSIS DF TLR +IS ++D +I E RMGI+VYS +A VP

Sbjct 28 GCKAQIDVVFVIDGSDSISESDFQTLRTSISRIVDGFHIGSGETRMGIIVYSKGVAFSVP 87

Query 88 LTSDKWTLQKEAEKLPHPRDGTNTALGIETMKELFQASPRPGVPKVGIVVTDGISKNTTE 147

L+ D L+ +A +PHPR+GTNT LGIE M ++F+ R GVP G+VVTDGISK +

Sbjct 88 LSYDPVYLKDQASIMPHPREGTNTHLGIEEMIDMFKKDKRDGVPMAGVVVTDGISKEKEK 147

Query 148 TVIQANLTKDLGINMFSVGVSQLIDMPELIGIASSERQVLTVDSFDALARNIEELVLLVC 207

T++Q+ L +DLGINMFSVGV + + EL GIAS+ Q + V+SFD L + + +LV LVC

Sbjct 148 TLLQSRLARDLGINMFSVGVGRYTEEEELRGIASNPDQAIKVESFDELLKILSKLVQLVC 207

Query 208 PTSPVPTTTMKPAPTPPPTQAPISGPCDKCKMQNGVGYNPHPDDCDKFTQCYFGGENQIM 267

P +KC M + Y P+D K + Y+ E +

Sbjct 208 P--------------------------NKCMMPGVIAY---PNDVSKNCRLYWKCEGE-- 236

Query 268 AVYRQCPFGQYWDQGMLTCRPSAEVNCPNDKCRHPRLLSYPYQGTTSCRAYWKCNFGKAI 327

+ LTC CP R + +F +

Sbjct 237 -------------ESKLTC-------CP--------------------RGF---SFSAPV 253

Query 328 GMCCPEGYEYNAFGGCVKSKKCNDTCPPQLINEGP-CDKRPSWKNEKGFEQYVYGFGWVD 386

C P+ KC + C +EGP C+KRPS +E+ + G+GWV

Sbjct 254 QSCIPD-------------PKCVEPCG----DEGPICNKRPSVYQPTIYEELIEGYGWVQ 296

Query 387 MPCAPGSRFDCNTCECSL-HTSFLPGRVCRPEVHIPFDKNCKDESGKAVYVENIDVTLTG 445

C PG+ +D TC C++ T P RVCR VHIPFD +C D SG ++N V T

Sbjct 297 RSCPPGTAYDRVTCGCTITQTPPPPRRVCRVLVHIPFDIDCVDTSGNGFLIKNHGVKFTR 356

Query 446 TGLGYFNGNTSKLIIPRFANVEHY--SDFVIKMRFMDV-TGGVRGMVSNGDCCSNEASLK 502

TGL F G +KL+IP N++ Y S+F++KMR+ + + +G++SNGDC + +L+

Sbjct 357 TGLALFEGK-AKLVIP---NIQRYLGSNFLVKMRYKEFPSFETQGLLSNGDCYT-PMTLQ 411

Query 503 MVKSERNIHYLAKCSRGATSTFYLPFIPGNWNEAYYMHDTKKLEGKVNGIGMEKWAYGSL 562

++K Y + S + F +P WNE + HD L G VNGI EK + G +

Sbjct 412 LIKDSIRHTYKIENSYRQRTGFTIPTNFKPWNEVTFSHDGNTLAGSVNGIQNEKRSNGPI 471

Query 563 RRSVYGPLKKSAAGLQIGFVKGFENFHGYMDYVTIYMC 600

+R+ Y G+ IGFV G+++F GYMDYVTIY C

Sbjct 472 QRTPY--------GISIGFVDGYKHFQGYMDYVTIYKC 501

>PfmPif97(Pinctada fucata martensii) unnamed protein product

Length=547

Score = 217 bits (553), Expect = 1e-066, Method: Compositional matrix adjust.

Identities = 145/538 (27%), Positives = 232/538 (43%), Gaps = 72/538 (13%)

Query 1 MQAITIKLAFFL---FFSGILTRVTLSEDSGCRSLIDFCIVVDGSDSISRDDFSTLRKAI 57

MQ +I++ F L F G+ + C++ D + VD SD +S DF L++A+

Sbjct 1 MQVPSIRVVFLLTAVFCVGVKS-------DECKT-ADVVVNVDASDDVSDQDFDKLKRAM 52

Query 58 SSLIDRLNIDKDEGRMGIVVYSSKIARKVPLTSDKWTLQKEAEKLPHPRDGTNTALGIET 117

++ L+ID ++ R+G+V Y S++ +PL D+ L + + P + G+

Sbjct 53 LMMVRGLSIDDNQIRLGMVTYGSEVCDSIPLQGDRLDLARTIRYMKKPTGPSKPFKGMGE 112

Query 118 MKELFQASPRPGVPKVGIVVT-DGISKNTTETVIQANLTKDLGINMFSVGVSQLIDMPEL 176

+ +F + R VP + + + D + + + + + +D I + ++G+ +D E+

Sbjct 113 ARRMFSSRGRYNVPHITMNLGGDIVDTEVKDLMDETDKARDEDIKVMAIGLGAKVDRDEI 172

Query 177 IGIASSERQVLTVDSFDALARNIEELVLLVCPTSPVPTTTMKPAPTPPPTQAPISGP--- 233

IA Q +D D L R ++E+ +C + P + +GP

Sbjct 173 ESIAYDRDQAYFMDDEDDLIRKVKEIPDYLCKIIKAKKPKVSGGKKSKPAKKVDNGPAGK 232

Query 234 -------------------------CDKCKMQNGVGYNPHPDDCDKFTQCYFGGENQIMA 268

CD + +GVGY P C+ F C +

Sbjct 233 SPGFDALKQSDDKSDKAKKVEVKELCDDAEWVDGVGYGSVPTRCEDFVMCQ-NVSGSLRK 291

Query 269 VYRQCPFGQYWDQGMLTCRPSAEVNCPNDKCRHPRLLSYPYQGTTSCRAYWKCNFGKAIG 328

+ CPFGQYW + +C + + +C +D C+ L S Y SCRAYWKC GK++

Sbjct 292 TLKSCPFGQYWSKRQTSCVLTEDEDCSDDLCKTMLLPSREYD--VSCRAYWKCEKGKSVA 349

Query 329 MCCPEGYEYNAFGGCVKSKKCNDTCPPQ----------------LINEGP-CDKRPSWKN 371

CCP G Y GCV C++ CPP+ I P C RP +

Sbjct 350 RCCPSGMAYEPGKGCVLDLDCDEECPPKNDGDDDDDSSDEDDDDEIEYNPNCPLRPIKGH 409

Query 372 EKGFEQYVYGFGWVDMPCAPGSRFDCNTCECSL----------HTSFLPGRVCRPEVHIP 421

+ F+Q+ W D CAPG+ F C CS+ +VC PE+++P

Sbjct 410 PEKFKQHTGDDNWEDFDCAPGTLFSARDCACSILGTAKKDDKNDDGGDAHKVCEPELYLP 469

Query 422 FDKNCKDESGKAVYVENI-DVTLTGTGLGYFNGNTSKLIIPRFANVEHYSDFVIKMRF 478

F + D SGK +VEN D + G YFNG L IPRF+ V + IKM++

Sbjct 470 FCDDLHDYSGKETHVENEGDAVIIENGKAYFNGRAG-LKIPRFSGVPYGKSVFIKMKY 526

>PpPif97(BAM66824.1Pteria penguin) unnamed protein product

Length=501

Score = 206 bits (525), Expect = 4e-063, Method: Compositional matrix adjust.

Identities = 146/488 (30%), Positives = 224/488 (46%), Gaps = 65/488 (13%)

Query 53 LRKAISSLIDRLNIDKDEGRMGIVVYSSKIARKVPLTSDKWTLQKEAEKLPHPRDGTNTA 112

+R+++S + L ID + +MG+VVY S + VPLT D+ TL K + P

Sbjct 1 MRRSLSMPVRGLGIDDIKTKMGVVVYGSSLGDSVPLTGDRMTLAKGIRNMKRPGGPGKPY 60

Query 113 LGIETMKELFQASPRPGVPKVGIVVTDGISKNT-TETVIQANLTKDLGINMFSVGVSQLI 171

+G+ TM+ +F++ R VPKV + I+ ET+ +A+ ++ I + ++GV + +

Sbjct 61 VGLNTMRSMFRSQARKNVPKVCLNFGRNIAPTVLKETLDEADKARNDDIKVVAMGVGRTV 120

Query 172 DMPELIGIASSERQVLTVDSFDALARNIEELVLLVCPTSPVPTTTMKPAPTP-------- 223

+ ++ IA S+ Q D L +NI + VC T+ P

Sbjct 121 EPDDVDNIAWSKGQAYRFSDEDDLMKNIRNIPEYVCSAIKANRPTLPQVPAANPPPAKPA 180

Query 224 ---------------PPTQAPISGPCDKCKMQNGVGYNPHPDDCDKFTQCYFGGENQIMA 268

++ P+ CD K +GVGY P C+KF CY G + +

Sbjct 181 SPQPKPDPQPKPKAVKESKPPVKELCDDAKWDSGVGYGSVPTRCEKFVMCYKGLGSSLRK 240

Query 269 VYRQCPFGQYWDQGMLTCRPSAEVNCPNDKCRHPRLLSYPYQGTTSCRAYWKCNFGKAIG 328

+ CPFGQ+W+Q + +C S + +C +D C++ L S Y S RAYWKC GK++

Sbjct 241 TIKTCPFGQFWNQMLSSCVRSEDEDCDDDPCKYNILPSRSYD--ISSRAYWKCENGKSVA 298

Query 329 MCCPEGYEYNAFGGCVKSKKCNDTCPPQL----------------------INEGP-CDK 365

CCP G Y + GC K ND CPP+ I+ P C

Sbjct 299 SCCPSGMAYKSGEGCYPDKDGNDDCPPKADSDNGEDVDDSSSSSSSEEEDDIDVNPNCPF 358

Query 366 RPSWKNEKGFEQYVYGFGWVDMPCAPGSRFDCNTCECSL--------------HTSFLPG 411

+P + F+Q++ WV CAPGS F C C+L +

Sbjct 359 KPVRGSNDKFKQHLGDDNWVTFDCAPGSLFSPLDCGCTLLDNGDDDDDSSSDEDDNDSEE 418

Query 412 RVCRPEVHIPFDKNCKDESGKAVYVENIDV-TLTGTGLGYFNGNTSKLIIPRFANVEHYS 470

CRP+V++PF KD SG++ ++N D + G+ YFNG +S L IPRF+ V +

Sbjct 419 ERCRPQVYLPFCDGLKDFSGRSTLIQNKDDGVVIKDGVAYFNGESS-LTIPRFSGVPYGD 477

Query 471 DFVIKMRF 478

VIKMR+

Sbjct 478 TVVIKMRY 485

>PmxPif97(BAJ08001.1Pinctada maxima) unnamed protein product

Length=561

Score = 204 bits (519), Expect = 1e-061, Method: Compositional matrix adjust.

Identities = 140/540 (26%), Positives = 229/540 (42%), Gaps = 74/540 (14%)

Query 8 LAFFLFFSGILTRVTLSEDSGCRSLIDFCIVVDGSDSISRDDFSTLRKAISSLIDRLNID 67

L L + + S+ + C++ D + VDGSD +S +F L++A+ L+ L+ID

Sbjct 6 LQIVLLLTAVFGIGVKSDSNDCKT-ADLVVSVDGSDDVSDREFDKLKRAMLMLVRGLSID 64

Query 68 KDEGRMGIVVYSSKIARKVPLTSDKWTLQKEAEKLPHPRDGTNTALGIETMKELFQASPR 127

+ R+G+V Y S I +PL D+ L + + P GI +++F + R

Sbjct 65 DSQIRLGMVTYGSDIGDSIPLQGDRLDLARTIRYMKKPGGPCKPFKGIGETRKMFSSRGR 124

Query 128 PGVPKVGIVV-TDGISKNTTETVIQANLTKDLGINMFSVGVSQLIDMPELIGIASSERQV 186

VP V + + D + + + + + +D I + ++G+ ++ E+ GIA + Q

Sbjct 125 FNVPHVTLNLGGDIVDSEVRDLMDETDKARDEDIKVLAIGLGAKVERDEIEGIAWDKDQA 184

Query 187 LTVDSFDALARNIEELVLLVCP-----------------------------TSPVPTTTM 217

+D D L R ++E+ +C SP +

Sbjct 185 YFMDDADDLVRRVKEIPDYLCKIIKAKKPRKSASKKSKTKPAKKPDSGLVGKSPGFHSLQ 244

Query 218 KPAPTPPPT-QAPISGPCDKCKMQNGVGYNPHPDDCDKFTQCYFGGENQIMAVYRQCPFG 276

+ P + + + CD + + VGY P C+ F C + + CP+G

Sbjct 245 QTDDKPKMSKKVEVKELCDDAEWVDDVGYGSVPTRCEDFVMCQ-NVSGTLRKTLKTCPYG 303

Query 277 QYWDQGMLTCRPSAEVNCPNDKCRHPRLLSYPYQGTTSCRAYWKCNFGKAIGMCCPEGYE 336

Q+W + +C + + +C +D C+ L S Y SCRAYWKC GK++ CCP G

Sbjct 304 QFWSRTRTSCILTEDEDCSDDLCKTMLLPSRDYD--VSCRAYWKCENGKSVARCCPSGMA 361

Query 337 YNAFGGCVKSKKCNDTCPPQ--------------------------LINEGP-CDKRPSW 369

Y GCV C++ CPP+ + P C +P

Sbjct 362 YEPGKGCVLDSDCDEECPPKDDSDNGDDGDDDDDDDDDEDDDDDEDEMEYNPNCPLKPIK 421

Query 370 KNEKGFEQYVYGFGWVDMPCAPGSRFDCNTCECSL----------HTSFLPGRVCRPEVH 419

+ + F+Q+ W + CAPG+ F C CS+ RVC PE++

Sbjct 422 GSPEKFKQHTGADNWEEFECAPGTLFSSRDCACSILGRPEKDDNGKNEDDTSRVCEPELY 481

Query 420 IPFDKNCKDESGKAVYVENI-DVTLTGTGLGYFNGNTSKLIIPRFANVEHYSDFVIKMRF 478

+PF + D SGK +VEN D + G YFNG L IPRF+ V + IKM++

Sbjct 482 LPFCDDLHDYSGKETHVENEGDAVIIENGKAYFNGRAG-LKIPRFSGVPYGKSVFIKMKY 540

>PmPif97(BAM66823.1Pinctada margaritifera) unnamed protein product

Length=558

Score = 202 bits (515), Expect = 3e-061, Method: Compositional matrix adjust.

Identities = 142/546 (26%), Positives = 233/546 (43%), Gaps = 77/546 (14%)

Query 1 MQAITIKLAFFLFFSGILTRVTLSEDSGCRSLIDFCIVVDGSDSISRDDFSTLRKAISSL 60

MQ +++ F L + + S+D C++ D + VDGSD +S +F L++A+ L

Sbjct 1 MQVPYLQIVFLL--TAVFGIGVKSDD--CKT-ADLVVNVDGSDDVSDREFDKLKRAMLML 55

Query 61 IDRLNIDKDEGRMGIVVYSSKIARKVPLTSDKWTLQKEAEKLPHPRDGTNTALGIETMKE 120

+ L+ID + R+G+V Y S+I +PL D+ L + + P GI ++

Sbjct 56 VRGLSIDDSQIRLGMVTYGSEIGDSIPLQGDRLDLARTIRYMKKPGGPCKPFKGIGETRK 115

Query 121 LFQASPRPGVPKVGIVV-TDGISKNTTETVIQANLTKDLGINMFSVGVSQLIDMPELIGI 179

+F + R VP V + + D + + + + + +D I + ++G+ ++ E+ GI

Sbjct 116 MFSSRGRFNVPHVTLNLGGDIVDSEVRDLMDETDKARDEDIKVMAIGLGTKVERDEIEGI 175

Query 180 ASSERQVLTVDSFDALARNIEELVLLVCP-----------------------------TS 210

A + Q +D D L R ++E+ +C S

Sbjct 176 AWDKEQAYFMDDADDLVRRVKEIPDYLCKIIKAKKPRKSASKKSKTKPAKKPDSDIVGKS 235

Query 211 PVPTTTMKPAPTPPPT-QAPISGPCDKCKMQNGVGYNPHPDDCDKFTQCYFGGENQIMAV 269

P + + P + + + CD + VGY P C+ F C +

Sbjct 236 PGFHSLQRTDDKPKMSKKVEVKELCDDAEWVEDVGYGSVPTRCEDFVMCQ-NVSGSLRKT 294

Query 270 YRQCPFGQYWDQGMLTCRPSAEVNCPNDKCRHPRLLSYPYQGTTSCRAYWKCNFGKAIGM 329

+ CP+GQ+W + +C + + +C +D C+ L S Y SCRAYWKC GK++

Sbjct 295 LKTCPYGQFWSRARTSCVLTEDEDCSDDLCKTMLLPSRDYD--VSCRAYWKCENGKSVAR 352

Query 330 CCPEGYEYNAFGGCVKSKKCNDTCPP--------------------------QLINEGPC 363

CCP G Y GCV C++ CPP ++ C

Sbjct 353 CCPSGMAYEPGKGCVLDSDCDEECPPKGDSDNGDDDDDDNDDDDNEYDDDDDEMEYNPNC 412

Query 364 DKRPSWKNEKGFEQYVYGFGWVDMPCAPGSRFDCNTCECSL----------HTSFLPGRV 413

RP + + F+Q+ W + CAPG+ F C CS+ +V

Sbjct 413 PLRPIKGSPEKFKQHTGDDNWEEFDCAPGTLFSSRDCACSILGRPEKDDNGKNEDDTSKV 472

Query 414 CRPEVHIPFDKNCKDESGKAVYVENI-DVTLTGTGLGYFNGNTSKLIIPRFANVEHYSDF 472

C PE+++PF + D SGK +VEN D + G YFNG L IPRF+ V +

Sbjct 473 CEPELYLPFCDDLHDYSGKETHVENEGDAVIIENGKAYFNGRAG-LKIPRFSGVPYGKSV 531

Query 473 VIKMRF 478

IKM++

Sbjct 532 FIKMKY 537

Lambda K H a alpha

0.321 0.138 0.440 0.792 4.96

Gapped

Lambda K H a alpha sigma

0.267 0.0410 0.140 1.90 42.6 43.6

Effective search space used: 1967684

Database: cgPif97.fa

Posted date: Mar 6, 2019 2:19 PM

Number of letters in database: 3,889

Number of sequences in database: 7

Matrix: BLOSUM62

Gap Penalties: Existence: 11, Extension: 1

Neighboring words threshold: 11

Window for multiple hits: 40
